# Editorial: Characterization of rare and recently first described human pathogenic bacteria

**DOI:** 10.3389/fcimb.2023.1212627

**Published:** 2023-07-03

**Authors:** Thomas Riedel, Boyke Bunk, Percy Schröttner

**Affiliations:** ^1^ Department of Microbial Ecology und Diversity Research, Leibniz Institute, German Collection of Microorganisms and Cell Cultures (DSMZ) GmbH, Braunschweig, Germany; ^2^ German Center for Infection Research (DZIF), Partner Site Hannover-Braunschweig, Braunschweig, Germany; ^3^ Department of Bioinformatics and Database, Leibniz Institute, German Collection of Microorganisms and Cell Cultures (DSMZ) GmbH, Braunschweig, Germany; ^4^ Carl Gustav Carus Faculty of Medicine, University Hospital Carl Gustav Carus Dresden, Institute for Medical Microbiology and Virology, Technical University (TU) Dresden, Dresden, Germany

**Keywords:** rare infections, bacterial pathogens, first description of bacterial species, editorial, comparative genomics, identification, epidemiology

The vast number of unknown bacteria is estimated to range between 10^7^ and 10^9^ (Overmann et al., 2017). Therefore, although routine clinical diagnostics are geared towards detecting the known human pathogenic bacteria, it becomes obvious that rare and even new species are observed and reported in clinical routine diagnostics as well. However, the knowledge of their clinical relevance often extends only to sporadic case reports. This confronts the medical microbiologist with considerable problems in individual case consultation, since this information alone is not sufficient to make a clear statement about the pathogenicity of the respective species and consequently whether they are causative for a disease or not. In certain clinical conditions (such as pre-existing immunosuppression) however, these species are nevertheless reported in many laboratories on findings, which also include a resistogram. Furthermore, it should also be noted that there is currently no standardized definition for “rare bacterial pathogens”. Following already existing definitions of rare diseases may therefore be the best approach to solve this dilemma. A common feature of the various definitions available is the use of the point prevalence for epidemiological assessment (Nguengang Wakap et al., 2020). According to a recent data analysis conducted by the “orpha-net” network (an association of 37 countries researching rare diseases), almost 6% of the world’s population is supposed to be affected by any kind of a rare disease (Nguengang Wakap et al., 2020). Transferring these findings to infectious diseases underlines the assumption that rare pathogenic bacterial species are indeed of clinical relevance. However, since rare human pathogenic bacteria have hardly been systematically researched so far, there is only very little information available on virulence, risk factors for infection, the clinical picture or the status of the patient (including immunosuppression). For this reason research into rare pathogens must be aimed be to work out which species has pathogenic potential for humans and which does not. It must also be clearly stated here that clinical case reports alone at best help to uncover circumstantial evidence, but ultimately cannot satisfactorily clarify the question of pathogenicity. Therefore, when starting to do research on rare pathogens, it is advisable to first establish a collection of the species of interest derived from clinical samples. Therefore, the first essential aspect to be clarified is the species identification strategy. The chosen method should ensure a high sample throughput while allowing reliable species identification. Based on our previous research, MALDI TOF MS currently seems to be the most suitable method for this purpose (Kostrzewa et al., 2019; Kopf et al., 2021; Bigge et al., 2022). In contrast, methods that use biochemical or metabolic traits for identification have not proven to be entirely reliable (Rudolph et al., 2019; Kopf et al., 2021; Bigge et al., 2022). The third option, which is commonly used in routine diagnostics, is the sequencing of the 16S rRNA gene. This method has also shown to produce reliable results, but is more time-consuming and requires specially trained personnel. For this reason, it is unsuitable for high-throughput examinations and therefore more applicable to confirm questionable identification results. In general, however, it should be noted that the identification accuracy of the respective methods varies for different species. Therefore, in order to determine the actual underlying species, genome-based methods such as the “digital DNA-DNA hybridisation” or calculation of the “average nucleotide identity” (ANI) should be applied (Richter and Rosselló-Móra, 2009; Rudolph et al., 2019; Kopf et al., 2021). The importance of genomic data for species identification has been addressed by several authors in this special edition. Reviewing the literature on the clinical significance of *Shewanella putrefaciens*, Müller et al. for instance pointed out that the species has frequently been misidentified in previous publications and the pathogenic species was assigned to *S. algae*. In addition, Wang et al. reported a patient with cervical carcinoma who died as a result of septic shock. Only with the help of genome sequencing, the authors were finally able to determine that the disease was caused by a yet unknown species of the genus *Peptoniphilus*. The species was subsequently designated as *Peptoniphilus septimus* sp. nov. Furthermore, Monecke et al. described staphylococcal strains from a straw-coloured fruit bat and a diamond firetail and gave a review of their phylogenetic relationships to other staphylococci. They propose that *Staphylococcus roterodami* and *Staphylococcus singaporensis* are distinct clonal complexes of the same species for which they propose the name *S. roterodami.* This species is also a known human pathogen. In addition, to further characterize rare pathogenic bacterial species, phenotypic examinations such as biochemical reactions or antimicrobial susceptibility profiles should be carried out, e.g. as done for initial species descriptions. For example, by comparing phenotypic results with genome data, the molecular basis of antimicrobial resistance can be determined. This approach can be used to describe and explain unusual resistance phenomena. Shittu et al. for instance described for the first time a *Staphylococcus argenteus* isolate in Germany that exhibited high resistance to mupirocin in addition to methicillin resistance. An increase of *S. argenteus* infections has been observed in recent years (Alhussein et al., 2020). In addition, Yao et al. described an *Enterobacter xiangfangensis* isolate that harbors both carbapenemases KPC-2 and OXA-48 and a mobile colistin resistance gene, which is an extreme rarity in this combination. Gaur et al. analyzed a *Chryseobacterium gallinarum* isolate that is highly resistant to colistin. In addition, the regional occurrence of rare species is important from an epidemiologic perspective. To date, there have been no systematic epidemiological studies on rare human pathogenic species. For this reason, it is currently still necessary to rely on the evaluation of published case reports to do this (Kopf et al., 2021). An important contribution to this Research Topic was made by Li et al. who were the first to report a bloodstream infection with *Herbaspirillum huttiense* in China. Of equal relevance is the initial description of previously unknown potential sources of infection. Zautner et al. for instance demonstrated that *Erysipelothrix rhusiopathiae* can be transmitted from animals (in this case pigs and dogs) to humans via bath water and can cause severe infections. Once a sufficient number of sequenced genomes of a species is available, it is advisable to analyze the pangenome using comparative genome analyses. Kopf et al. have impressively demonstrated this for *Wohlfahrtiimonas chitiniclastica*. In addition to the direct investigation of rare isolates, it is important to place the topic of rare human pathogenic bacteria in a broader clinical context in order to understand their significance to various diseases. For example, Felber et al. showed in a retrospective study of appendicitis in pediatric patients that there is no significant difference in complication rates when rare pathogens are detected. However, future prospective multi-centric studies including significantly more patients and isolates will provide more clarity.

## Author contributions

All authors listed have made a substantial, direct, and intellectual contribution to the work and approved it for publication.
